# Social Distancing with Older Adults: Comparing Pre- and Post-Pandemic Preferences in Children

**DOI:** 10.1093/geroni/igaa057.3500

**Published:** 2020-12-16

**Authors:** Juliana Lapp, Jenny Bauer, Lea Scholz, Sonja Steltmann, Marit Lange, Lena-Emilia Schenker, Jennifer Bellingtier

**Affiliations:** 1 Friedrich Schiller University Jena, Jena, Germany; 2 Friedrich Schiller University Jena, Jena, Thuringen, Germany

## Abstract

The Covid-19 pandemic has made age more salient, and the media has included numerous ageist messages (Bronwen, 2020), included messages aimed at children (e.g., “stay home to protect grandma and grandpa!”). When the pandemic reached Germany in March, we halted data collection on a project assessing ageism in children ages 4 to 8. In July, the situation had improved and testing resumed following hygiene protocols. We report findings from a simulated-behavioral measure where 45 children were asked to plan a party. One task involved asking the children to place pictures of ten party guests, plus themselves, around two party tables. We then assessed how many seats away the younger and older adult guests were placed, on average, from the child (i.e., social distance). Although, we anticipated that the pandemic might lead children to further distance themselves from older adults, our results, thus far, indicate similarity between pre- and post-pandemic preferences. At both occasions older adult guests were seated, on average, one seat further away from the child then younger adult guests. The guest chosen to sit closest to the child was younger on 88% of occasions, whereas the guest chosen to sit farthest away from the child was older on 64% of occasions. Preference for younger adults was confirmed in a second task where children selected teammates for a game. On average, children’s teams consisted of 70% younger guests versus 30% older guests. Findings indicate a social preference for younger, versus older, adults in children irrespective of the pandemic.

